# All routes lead to Rome: multifaceted origin of hepatocytes during liver regeneration

**DOI:** 10.1186/s13619-020-00063-3

**Published:** 2021-01-06

**Authors:** Ce Gao, Jinrong Peng

**Affiliations:** MOE Key Laboratory for Molecular Animal Nutrition, College of Animal Sciences, Zhejiang University, Hangzhou, 310058 China

## Abstract

Liver is the largest internal organ that serves as the key site for various metabolic activities and maintenance of homeostasis. Liver diseases are great threats to human health. The capability of liver to regain its mass after partial hepatectomy has widely been applied in treating liver diseases either by removing the damaged part of a diseased liver in a patient or transplanting a part of healthy liver into a patient. Vast efforts have been made to study the biology of liver regeneration in different liver-damage models. Regarding the sources of hepatocytes during liver regeneration, convincing evidences have demonstrated that different liver-damage models mobilized different subtype hepatocytes in contributing to liver regeneration. Under extreme hepatocyte ablation, biliary epithelial cells can undergo dedifferentiation to liver progenitor cells (LPCs) and then LPCs differentiate to produce hepatocytes. Here we will focus on summarizing the progresses made in identifying cell types contributing to producing new hepatocytes during liver regeneration in mice and zebrafish.

## Background

From an ancient myth to nowadays a norm, the concept of liver regeneration has fascinated human society for over hundred years. Being the only visceral organ that can regain its original mass through compensatory growth after partial hepatectomy (PH) or exposure to toxins, liver regeneration has been attracting researchers in two main aspects: 1) the biology of liver regeneration, 2) the application in treating human liver diseases. In both aspects, tremendous achievements have been achieved and have been reviewed in many excellent reviews (Fausto et al., 2006; Milne, 1909; Liu & Chen, 2017; Michalopoulos, 2014; Cox & Goessling, 2015; Stanger, 2015; Miyajima et al., 2014; Michalopoulos, 2017; Taub, 2004; Michalopoulos, 2010; Michalopoulos, 2013; Michalopoulos, 2007; Abu Rmilah et al., 2019; Mao et al., 2014). Regarding the biology of liver regeneration, the main goals include to understand the genetic and molecular control of liver regeneration and the origin of new hepatocytes in the regenerated liver. In recent years, with the help of newly developed lineage tracing tools, many efforts have put on determining the origin of new hepatocytes during liver regeneration. Surprisingly, researchers noticed that new hepatocytes can be derived from a variety of cell types, suggesting that nature has evolved versatile ways to maintain the liver function. In this review, we will mainly focus on summarizing the progresses made in identifying cell types contributing to produce new hepatocytes during liver regeneration in mice. We will also discuss the perspective of the use of zebrafish as a model in studying liver regeneration.

## Approaches for creating liver-damage models

Various approaches have been applied to study the process of liver regeneration. The first and also most drastic approach is to resect a portion (up to 70%) of the liver lobes (Bohm et al., 2010). The competency of liver regeneration after resection (partial hepatectomy, or PH) is the prerequisite for liver transplantation. This approach has been well established in human, rat, mouse and zebrafish. The main obstacle for this approach is how to stop bleeding after operation.

The second approach is to use chemicals as listed below to cause liver damage or kill hepatocytes and then follow the process of liver regeneration.

### CCl_4_ (carbon tetrachloride)

CCl_4_ induced liver injury is usually performed by intraperitoneal injection. CCl_4_ is activated in hepatocytes and forms the trichloromethyl radical, which results in serious damage to hepatocytes, such like the loss of cellular calcium sequestration and homeostasis. CCl_4_ also activates TNFα, TGFα and TGFβ pathways in the cell, processes that appear to direct the cell primarily toward (self-)destruction or fibrosis (Weber et al., 2003).

### DDC (3,5-diethoxycarbonyl-1,4-dihydrocollidine)

DDC feeding results in a phenotype of active secretion of porphyrin by BECs (biliary epithelial cells) and persistent proliferation of primitive ductules with poorly defined lumens in mice, which leads to cholestatic liver injury (Fickert et al., 2007; Preisegger et al., 1999).

### CDE diet (choline-deficient, ethionine-supplemented diet)

feeding mice with CDE diet results in a physiologically relevant model of liver disease which mimics the human condition of chronic fatty liver disease (Akhurst et al., 2001; Passman et al., 2015). Ethionine has antagonistic activity against methionine which enables it to interfere with methylation metabolism to cause liver damage.

### NTR/Mtz system

*Escherichia coli* enzyme nitroreductase (NTR) can efficiently catalyze the oxygen-independent reduction of nontoxic prodrugs, such as 1-(2-hydroxyethyl)- 2-methyl-5-nitroimidazole (Mtz), and converts the prodrugs into a cytotoxic agent, which causes DNA interstrand cross-linking and cell death (Curado et al., 2007). Curado and his colleagues generated transgenic fish *Tg(l-fabp: CFP-NTR)*^*s891*^ which specifically expressed NTR protein in zebrafish hepatocytes. NTR protein has no effect on the hepatocytes itself, so conditional hepatocytes ablation model can be created by the Mtz exposure in this transgenic fish (Curado et al., 2007).

### APAP (acetaminophen (N-acetyl-p-aminophenol)

APAP is one of the most commonly used medications to alleviate pain and fever, and unfortunately has serious side effects under certain conditions, which could cause severe liver damage (Goessling & Stainier, 2016). Goessling and colleagues reported an adult zebrafish liver injury model based on APAP exposure (North et al., 2010). Further research on this injury model found that inhibition of the enzymatic regulator S-nitrosoglutathione reductase (GSNOR) can protect liver from toxic damage and increase cell proliferation. This result indicated that the APAP-GSNOR system might be able to be adopted to study liver regeneration.

### AA (allyl alcohol)

AA can be absorbed immediately after injection by the liver parenchymal cells lying at the beginning of the sinusoids, which cause hepatocellular damage in the periportal portion of the liver lobule (Sasse & Maly, 1991). The toxicity of AA is due to oxidation of AA by liver alcohol dehydrogenase to acrolein, a highly reactive electrophilic metabolite, which can trigger cellular necrosis (Badr, 1991).

## Origin of new hepatocytes during mice liver regeneration

It has been widely appreciated that liver regeneration after PH in a normal animal is achieved mainly through cell cycle reentry of existing hepatocytes although the mechanism for controlling the scarless healing of the resection site remains enigma (Overturf et al., 1997; Yanger et al., 2014). However, as summarized in the following, many reports have shown that acute or chronic liver damage induced by toxic drug/chemicals, cell ablation or diseases appears to activate different categories of cells to participate in liver regeneration.

### Oval cells (EpCAM^*+*^*)*

In the 1956, Farber and colleagues found a kind of small and undifferentiated hepatic epithelial cells and named them “oval cells”, because of their ovoid nucleus (Farber, 1956). Later studies showed that “oval cells” are EpCAM^+^ (Okabe et al., 2009). DDC diet caused liver injury can induce oval cells (EpCAM^+^) activation characterized by significant increase of proliferation of EpCAM^+^ cells after DDC treatment. In addition, injury led to the specific expression of marker genes (such as TROP2 and Folx1) in oval cells, which were hardly detected in normal liver (Okabe et al., 2009; Sackett et al., 2009). In vitro experiments showed that EpCAM^+^ cells, which isolated from injured liver, could proliferate to form colonies and differentiate into hepatocytes (Alb^+^, Afp^+^) and biliary epithelial cells (Ck7^+^, Ck19^+^) (Okabe et al., 2009; Suzuki et al., 2008).

Some researchers isolated oval cells and transplanted them to FAH-deficient (FAH^−/−^) recipient mice to examine the differentiation potential and self-renewing capability of these cells (Suzuki et al., 2008; Wang et al., 2003). They found that the transplanted clonogenic progeny of oval cells could give rise to morphologically and functionally mature hepatocytes in recipient mice (Suzuki et al., 2008).

### Parenchymal hepatocytes

Comprehensive experiments indicated that virtually all new hepatocytes come from preexisting hepatocytes in mice, not only in liver growth and homeostasis but also in the regeneration process (Yanger et al., 2014; Schaub et al., 2014). After more than 99% of hepatocytes were lineage labeled, the AAV8-TBG-Cre injected *R26*^*YFP*^ mice were used to study liver regeneration under different liver damage conditions including drug/chemical treatment (DDC, CDE, CCl_4_, ANIT) and PH. Under these conditions, the percentage of labeled hepatocytes remained unchanged, which means few hepatocytes arise from non-hepatocytes after liver regeneration (Yanger et al., 2014). However, it is now commonly appreciated that parenchymal hepatocytes are highly heterogeneous, it is necessary to determine which subtype of parenchymal hepatocytes are mobilized during liver regeneration after liver damage.

#### Telomerase^High^ hepatocytes

Previous studies show that cell renewal is dependent in part on the synthesis of telomere repeats (Batista & Artandi, 2013). Lin and colleagues designed a TertCreERT2^/+^ knock-in mouse strain, which carried CreERT2 downstream the telomerase reverse transcriptase (Tert) locus (Lin et al., 2018). They found that rare hepatocytes with high telomerase expression are distributed throughout the liver lobule. During homeostasis or recover from injuries, the progeny of TERT^High^ hepatocytes expands throughout the lobule, which provides the preliminary experimental evidence for the renewal capacity of the hepatocytes in any lobular zone.

#### Periportal Sox9^low+^ hybrid hepatocytes

A study reported that there is a pre-existing population of periportal hepatocytes which could make major contribution to hepatocytes regeneration after chronic liver damage. These hybrid hepatocytes located in the portal triads of healthy livers, and expressing low amounts of Sox9 and other bile-duct-enriched genes (Font-Burgada et al., 2015). The authors treated Sox9-CreERT mice with two weekly CCl_4_ injections for 6 weeks, and found that the progeny of labeled cells expanded and made significantly contribution to parenchymal hepatocytes restoration, comparing with acute CCl_4_ treatment group (Font-Burgada et al., 2015). In addition, the transplanted Sox9 positive hybrid hepatocytes in FAH-deficient (FAH−/−) recipient mice shows better proliferation capacity than conventional hepatocytes (Font-Burgada et al., 2015).

#### Sox9^+^ liver progenitor-like cells (LPLC)

Li and colleagues found that DDC treatment stimulates partial hepatocytes to express both Hnf4a and liver progenitor markers Sox9, Opn, and CD24 (Li et al., 2019). To reveal whether these hepatocytes make contribution to regeneration, they treat Sox9-CreERT2 with both DDC diet and Tamoxifen for 2 weeks. The results showed that around 24.2% of hepatocytes were derived from the labeled hepatocytes (Sox9^+^ during treatment) in regenerated livers, demonstrating that this subtype of parenchymal hepatocytes is one source of cells contributing to liver regeneration (Li et al., 2019).

#### Axin2^+^ hepatocytes around central vein

Axin2 is a universal transcriptional target of β-catenin-dependent Wnt signaling. In the adult liver, Axin2 is expressed in cells located around the central vein because Wnt2 and Wnt9b were expressed exclusively in central vein endothelial cells (Wang et al., 2015). Long term lineage tracing experiments based on Axin2-CreERT mouse indicated that labeled hepatocytes proliferate faster than other ones and can replace all hepatocytes along the adult liver lobule during homeostatic renewal (Wang et al., 2015). The labeled hepatocytes are diploid and express Tbx3, a transcription factor important in maintaining pluripotency (Wang et al., 2015).

However, this concept is challenged by two recent reports. Considering the fact that the insertion of the CreERT2 cassette disrupted one Axin2 allele in this transgenic line, (van Amerongen et al., 2012) which may cause haploinsufficiency of Axin2, (Sun et al., 2020). Sun and colleagues developed BAC-transgenic Axin2-CreERT2 mice without heterozygous deletion of Axin2 so that to exclude the possibility that potential haploinsufficiency of Axin2 might bias proliferation of Axin2 positive hepatocytes (Sun et al., 2020). Indeed, unlike previous studies, although the number of EGFP-labeled pericentral hepatocytes increased between 1 and 7 days after Tamoxifen treatment, no further increase in the number of lineage labeled hepatocytes from day 7 to 10 months were observed (Sun et al., 2020). This result suggests that there is no superior renewal capacity to the Axin2 positive pericentral vein hepatocytes. EdU and Ki67 antibody staining experiments also confirmed that EdU incorporation and Ki67 positive signal is equal in all liver zones during liver regrowth following PH (Sun et al., 2020).

By using AAV8-TBG-Cre and heterozygous Rosa26-Rainbow Cre reporter mice, single hepatocytes were randomly labeled (Chen et al., 2020a). After 13 months, they found that 90.1% were single cells, 9.0% were 2-cell clones, 0.7% were 3- to 4-cell clones, and only 0.2% consisted of 5–7 cells. It is noteworthy that clones > 2 cells were mostly located in the midlobular zone while pericentral and periportal clones almost exclusively consisted of 1 or 2 cells. After the pericentral hepatocytes were specifically injured by intraperitoneal injection of CCl_4_, they saw compensatory proliferation of hepatocytes in periportal area. And when the periportal hepatocytes were specifically injured by AA, compensatory proliferation of hepatocytes were localized in pericentral area (Chen et al., 2020a). After 12 doses of CCl4, periportal hepatocytes made a major contribution to restore hepatocytes, some of which covered the distance from the portal vein to the central vein. This result is consistent with the observation by Font-Burgada and colleagues (Font-Burgada et al., 2015).

#### Polyploidy hepatocytes

Based on the Cre-loxP system, Matsumoto and colleagues developed a multicolor reporter allele system to genetically label and trace diploid and polyploid cells in situ (Matsumoto et al., 2020*)*. In the Rosa-Confetti multicolor reporter mouse, Rosa-Confetti allele consists of a floxed stop cassette followed by four different reporter genes (Snippert et al., 2010). After Cre recombination, diploid cells can express only one reporter gene, whereas polyploids can be labeled by co-expressing multiple colors in germline heterozygous Rosa-Confetti mice (Matsumoto et al., 2020). Clonal tracing experiments shows that polyploid hepatocytes have extensive proliferation capacity in Fah transplantation model or after injuries including DDC, TAA, CCl_4_ (Matsumoto et al., 2020).

### Sox9^+^ and Krt19^+^ bile duct cells

Mature hepatocytes and biliary epithelial cells are both derived from hepatoblasts (Zong & Stanger, 2012). Therefore, it is not surprising that hepatocytes can undergo hepatocyte-to-BEC reprogramming following different kinds of liver injuries including DDC treatment, bile duct ligation and partial hepatectomy (Yanger et al., 2013). Vise versa, studies have shown that biliary epithelial cells could give rise to hepatocytes in certain circumstances. Cell transplantation assays indicated that biliary-epithelial-like cells have potential to give rise to both hepatocytes and BECs during DDC-mediated liver injury. However, it appears that this potential is quite limited and dependent on the physiological state of hepatocytes (Yanger et al., 2014; Schaub et al., 2014; Yanger et al., 2013; Malato et al., 2011; Rodrigo-Torres et al., 2014; Tarlow et al., 2014).

Since *sox9* is a widely recognized biliary marker, many cell lineage tracing experiments were performed on Sox9-CreERT2 (or Sox9-IRES-CreERT2) mouse (Carpentier et al., 2011; Furuyama et al., 2011). In the long-term (~12mth) chase experiments with 5 times tamoxifen treatment, it was observed that the continuous hepatocyte supply was from the Sox9-lineage-labeled precursor (Furuyama et al., 2011). So, the authors concluded that biliary epithelial cells made contribution to the physiological homeostasis of hepatocytes (Furuyama et al., 2011). In addition, tissue section analysis and FACS analysis indicated that Sox9 lineage-labeled cells may give rise to a fraction of adult hepatocytes after DDC injury treatment (Carpentier et al., 2011; Dorrell et al., 2011).

However, this concept is challenged by the fact that Sox9 and some other biliary cell genes expression could be induced in hepatocytes by tamoxifen administration or under pathological conditions (Yanger et al., 2013; Tarlow et al., 2014; Carpentier et al., 2011). Therefore, Yanger and colleagues used Krt19-CreERT and the thymidine analogs iododeoxyuridine (IdU) and chlorodeoxyuridine (CldU) to label biliary epithelial cells (Yanger et al., 2014). By using HNF4a antibody as hepatocyte marker, they found that the labeled cells make almost no contribution to hepatocytes during regeneration from injuries including DDC or CDE treatment.

Some subsequent reports suggested that the contribution by Sox9^+^ and Krt19^+^ bile duct cells after liver damage depends on the genetic or pathophysiological status of hepatocytes. For example, Raven and colleagues found that impairing hepatocyte proliferation by hepatocyte ablation of *β1-integrin* or overexpression of p21 induced cholangiocytes to form hepatocytes after liver injury in mouse (Raven et al., 2017). Similarly, Russell and colleagues found that, following CDE diet–induced liver injury, *β-catenin* knockout in *Ctnnb1*^*flox/flox*^ mice with AAV8-TBG-Cre injection induced a conversion of biliary epithelial cells to hepatocyte (Russell et al., 2019a). Deng and colleagues reported that long-term (more than 24 weeks) thioacetamide or DDC treatment will lead to significant expansion of CK19^CreERT^ labeled HNF4α^+^ cells in AAV^Cre^ injected CK19^CreERT^ mice (Deng et al., 2018). Similarly, Manco and colleagues found that the conversion of cholangiocytes (labeled by osteopontin-iCre^ERT2^) into hepatocytes could occupy 12% of the liver parenchyma by week 8, however, reduced to 5% by week 16, after treatment by CCl_4_ for >24weeks (Manco et al., 2019). These results demonstrated that chronic liver injury under specific genetic or pathophysiological conditions will induce conversion of biliary epithelial cells into hepatocytes (Deng et al., 2018).

## Zebrafish as a model for studying liver regeneration

Zebrafish liver has similar anatomical structures and cellular components to those in a mammalian liver (Goessling & Stainier, 2016; Korzh et al., 2008). The ease of performing liver amputation and quick regeneration of the amputated liver, together with its genetic advantages, makes the zebrafish an ideal model for studying liver regeneration. The earliest report on using zebrafish as a model organism for studying liver regeneration was published on 2007 (Sadler et al., 2007). Since then, more than 20 research papers related to these topics have been published. Prior to summarizing these progresses, it is necessary to introduce the current understanding of the molecular and cellular control of liver organogenesis in zebrafish.

### Origin of hepatocytes during liver growth and homeostasis in zebrafish

As in mice, zebrafish liver initiation and morphogenesis are regulated by signalling factors such as bone morphogenesis protein (BMP), fibroblast growth factor (Fgf), Wnt etc (Wang et al., 2017). In mice, BMP signals are secreted from the septum transversum mesenchyme, FGF signals come from cardiac cells, (Palaria et al., 2018) and Wnt signals come from mesodermal layer (Zakin et al., 1998; Si-Tayeb et al., 2010). Correspondingly, in zebrafish, BMP signals are secreted from lateral mesoderm, (Chung et al., 2008; Huang et al., 2008). FGF signals come from mesenchyme cells,(Dong et al., 2007) and Wnt signal comes from lateral plate mesoderm directly adjacent to the liver-forming endoderm (Ober et al., 2006). Following liver budding, hepatoblasts differentiate into hepatic parenchymal cells and bile duct cells. This process is regulated by several specific transcription regulators including Hhex, (Hunter et al., 2007; Gao et al., 2019), Prox1, Hnf4α, Sox17 (Field et al., 2003; Spence et al., 2009) and Sox9 (Furuyama et al., 2011; Delous et al., 2012) etc. Interestingly, the expression patterns of these transcription factors are also highly conserved between mice and zebrafish.

An important prerequisite for studying liver regeneration is to understand the origin and source of hepatic parenchymal cells in an adult liver. Take the advantage of the zebrafish genetic model, Gao and colleagues generated a transgenic fish that allowed to label the embryonic hepatocytes permanently. Their lineage tracing experiments revealed that although the number of hepatocytes in adult zebrafish was 1000 times more than that in embryos at 5 days post fertilization (dpf) the increased hepatocytes were almost solely derived from the embryonic hepatocytes. In addition, the homeostasis of hepatocytes appears to be maintained through proliferation of mature hepatic parenchymal cells (Gao et al., 2018). Although the origin of hepatocytes during liver growth in mice has not been well established the current evidence shows that the homeostasis of hepatocytes in adult mice appears to be maintained through proliferation of liver parenchymal cells (Yanger et al., 2014).

### Liver regeneration after PH

The liver of an adult zebrafish consists of three hepatic lobes: ventral lobe, left and right dorsal lobes. The junction of the three lobes is located in the anterior of the abdominal cavity (Korzh et al., 2008). The ventral hepatic lobe of zebrafish is very close to the ventral abdominal wall and has clear morphological characteristics, which is conducive to the implementation of PH and provides favorable congenital conditions for the study of regeneration after PH.

Performing PH in zebrafish PH is comparatively simple (Goessling & Stainier, 2016). Studying zebrafish liver regeneration via the PH approach was first tried in 2007 (Sadler et al., 2007). Since then, seven reports in the content of studying zebrafish liver regeneration via PH have been published. The work by Kan and colleagues described systematically the compensatory regeneration after abdominal lobectomy in zebrafish (Kan et al., 2009). They found that, after the ventral lobes were almost entirely resected, liver regeneration occurs via compensatory growth mediated by proliferation of hepatocytes throughout the entire liver remnant in 7 days (Kan et al., 2009). The authors also proposed that the liver-to-body ratio (LBR) is a relatively reliable criterion for evaluating liver regeneration (Kan et al., 2009).

Interestingly, it was noticed that different degrees of hepatic lobectomy may follow different regeneration mechanisms. For example, the zebrafish ventral lobe can fully regenerate after resecting the tip of the ventral lobe (Kan et al., 2009; Goessling et al., 2009). The mechanism behind this phenomenon is still unclear. Experiments in mice have proved that shear stress plays a role in regeneration initiation and homeostasis regulation (Lorenz et al., 2018; Sato et al., 1999; Schoen et al., 2001). Therefore, different degrees of resection will inevitably lead to different levels of blood flow shear stress changes, which may be the reason for the difference in regeneration mode (Lorenz et al., 2018).

Several genes, when in halpoinsufficiency (i.e heterozygous mutant), have been found to be involved in regulating liver regeneration/regrowth after PH. For example, *uhrf1* (a gene participating in DNA methylation and cell cycle regulation) (Sadler et al., 2007) and *top2a* (a gene involved in chromosome decatenation) (Dovey et al., 2009) heterozygous mutants both show defects in liver regrowth after PH, likely due to their role in mediating hepatocytes cell cycle progression. Recently, Chen et al. found that depletion of Calpain 3b (Capn3b), a Ca2 + −dependent cysteine proteinase, delayed liver regeneration by disrupting the synchronization of cell cycle reentry during liver regeneration, probably by accumulating G2/M transition inhibitors Chk1 and Wee1 (Chen et al., 2020b).

Kan and colleagues found that expression of dnBMPR or dnFGFR1 (dn: dominant negative) leads to defect on liver mass recovery after PH (Kan et al., 2009). Goessling and colleagues found that the PGE2/wnt interaction may act as a central regulator during dorsal lobe regeneration after partial hepatectomy. Hepatocytes proliferation will be unregulated in the *wnt* enhanced zebrafish Apc^+/−^. In addition, inhibition of Prostaglandin E2 (PGE2) by indomethacin exposure significantly diminished the number of proliferating hepatocytes both in wild type fish and *apc*^*+/−*^ mutant (Goessling et al., 2009).

### Liver regeneration at the amputation site in zebrafish

As in mammals, zebrafish liver regeneration after PH is through compensatory growth. However, a rarely discussed issue is how the amputation site is healed without leaving an obvious scar. Zhu and colleagues found that inflammatory response is activated immediately after PH which will help to cleanse the apoptotic cells at the amputation site, thus to facilitate wound healing by forming a fibril layer. Between 6 and 36 h post PH, the pioneer neutrophil will be removed by macrophages. At 5dpH, the amputation site will undergo remodeling and finish liver regeneration without leaving a scar (Zhu et al., 2014). The *digestive organ expression factor* (*def*) gene encodes a nucleolar protein essential for the ribosome small subunit biogenesis (Chen et al., 2005; Tao et al., 2013; Guan et al., 2016; Zhao et al., 2019). Through studying the *def*^*+/−*^ heterozygous mutant, Zhu and colleagues found that *def*^*+/−*^ heterozygous mutant suffered from a prolonged inflammatory response in the liver, including the amputation site, after PH. The constant inflammatory reaction prevents the resolving of the fibril layer which finally results in fibrosis at the amputation site (Zhu et al., 2014). In the end, although the LBR is recovered in *def*^*+/−*^ heterozygous mutant at 7dpH fibrosis results in abnormal scar formation (Zhu et al., 2014). Mechanistically, *def*^*+/−*^ halpoinsuffciency leads to p53 accumulation. Accumulated p53 promotes the expression of HMGB1 that in turn activates the inflammatory response. Consequently, Constant inflammatory response activates TGF-β signaling to cause fibrosis at the amputation site (Zhu et al., 2014).

### Transdifferentiation of bile duct cells after ablation of hepatocytes

Using the NTR/Mtz system, Shin and Luo two labs independently ablated almost all hepatocytes in 5dpf zebrafish embryos (Choi et al., 2014; He et al., 2014). The zebrafish embryos survived under this situation. Combining with Cre/loxp genetic cell lineage tracing system, both labs found that such embryos regenerated their liver through transdifferentiation of biliary epithelial cells (BECs) to mature hepatocytes during regeneration, and this process is regulated by *wnt2bb* and *sox9b* (Choi et al., 2014*;* He et al., 2014*)*. It is a breakthrough finding in liver progenitor cells study field: before that, there are a lot of reports talking about the transdifferentiation capacity of the BEC in vitro, but only few reports provide solid evidences in vivo (Tarlow et al., 2014).

From 2014 to 2020, about 11 research articles on zebrafish liver regeneration were published using the NTR/Mtz system. Based on these studies, the process of liver regeneration can be outlined into three stages: BEC dedifferentiation, liver progenitor cells (LPC) differentiation, hepatocytes and BEC proliferation.

He and colleagues found that mTOR1 signaling is required for the dedifferentiation of BECs to LPCs after liver injury (He et al., 2019). They found that mTORC1 signaling was upregulated in BECs during extreme hepatocytes ablation and continuously expressed in later liver regeneration. Early mTORC1 signaling inhibition, either by chemical or genetics, will severely block the dedifferentiation of BECs to HPCs. In this study, the authors also found that inhibition after liver injury reduced the proliferation of LPCs derived hepatocytes. This hypothesis is confirmed by a recent research showing that that E2 (17β-estradiol) promoted zebrafish liver regeneration via activation of the GPER1 and mTORC1 pathways (Chaturantabut et al., 2019).

Studies by Choi and colleagues showed that Bmp signaling regulates LPCs differentiation into hepatocytes through Tbx2b and BEC proliferation through Id2a, suggesting that the key developmental signaling pathways of liver are reused during liver regeneration (Choi et al., 2014). Ko and colleagues found that BET (Bromodomain and extraterminal domain) inhibitor can impair BEC-driven liver regeneration at multiple steps, including BEC dedifferentiation, HB-LC proliferation, hepatocytes proliferation, and hepatocyte maturation (Ko et al., 2016). Furthermore, Notch signaling also plays a role in LPCs differentiation regulation during liver regeneration. By studying on epigenetic factor Hdac1, Ko identified Notch3 as the receptor that regulates differentiation of LPCs into BECs (Ko et al., 2019). Sox9b (Sox9 homolog protein in zebrafish) is a direct target of Notch signaling and is required for BEC morphogenesis (Manfroid et al., 2012). Inhibition of the Notch-Sox9 signaling axis promotes LPC-to-hepatocyte differentiation in LPC-mediated liver regeneration (Russell et al., 2019b). On the other hand, LPC-to-hepatocyte differentiation was enhanced by EGFR inhibitor (AG1478) treatment, in which Sox9b might also play a role as a key downstream effector (So et al., 2020). In addition, Sox9b suppresses LPC-to-hepatocyte differentiation cell-autonomously in *Tg (fabp10a:CreERT2;ubb:loxP-GFP-loxP-sox9b-2A-mCherry)* and *Tg (fabp10a:CreERT2;ubb:loxP-GFP-loxP-dnsox9b-2A-mCherry)* lines (So et al., 2020). Based on these results, it appears that Sox9b regulates the process of LPC-to-hepatocyte differentiation through both the Notch-Sox9 and EGFR-ERK-SOX9 pathways. Khaliq and colleagues found that Stat3/Socs3a pathway is necessary for the proper timing of LPC-to-hepatocyte differentiation and establishing the proper number of BECs during LPC-driven liver regeneration (Khaliq et al., 2018). It is worth to mention that an RNAseq analysis of the regenerating liver at 0 h, 12 h, 24 h, 4 days, 6 days, and 8 days post NTR/Mtz system injury failed to identify the gene signatures of dedifferentiation of BECs to LPC. The authors speculated that could be resulted from moderate rather severe liver damage after Mtz treatment (Jagtap et al., 2020).

Several of the above reports used small molecule inhibitors in their studies, such as Hdac1 inhibitor MS-275, BET inhibitor iBET151 and JQ1 (Ko et al., 2016; Ko et al., 2019). These small molecules were identified in chemical screening for promoting/inhibiting liver progenitor cell-driven liver regeneration in the NTR/Mtz system mediated liver damage model (Ko & Shin, 1905). In this model, the authors used *Tg (fabp10a:CFP-NTR);Tg (fabp10a:DsRed)* double transgenic fishes to achieve a visible and high throughout system which allows for screening ~ 30 compounds per week (Ko & Shin, 1905).

### Hepatocytes and bile duct cells co-contribution

Curado and his colleagues reported a zebrafish *tomm22* gene mutant which suffered from specific hepatocyte apoptosis (Curado et al., 2010). The authors also noticed that Tomm22 transient knockdown reduced the number of hepatocytes at 5dpf, however, followed by a fully recovered liver at 8dpf (Curado et al., 2010). Further study using cell lineage system showed that temporary knockdown of *tomm22* not only activated BECs to give rise to hepatocytes but also induced the phenotypic change of surviving hepatocytes, generating hybrid hepatocytes (Wu et al., 2017). This hepatocyte conversion and redifferentiation in zebrafish seems not a special case, in mouse liver regeneration research, under chronic injury model (12 times CCl_4_ treatment in 6 weeks), Sox9^low+^ hybrid hepatocytes were found to make mainly contribution to the new born hepatocytes (Font-Burgada et al., 2015). In addition, hepatocytes can convert into peripheral cholangiocytes in Alb-cre^+/−^Rbpj^f/f^Hnf6 ^f/f^ mice which lack peripheral bile ducts at birth (Schaub et al., 2018).

## Summary and prospect

**Fig. 1:**
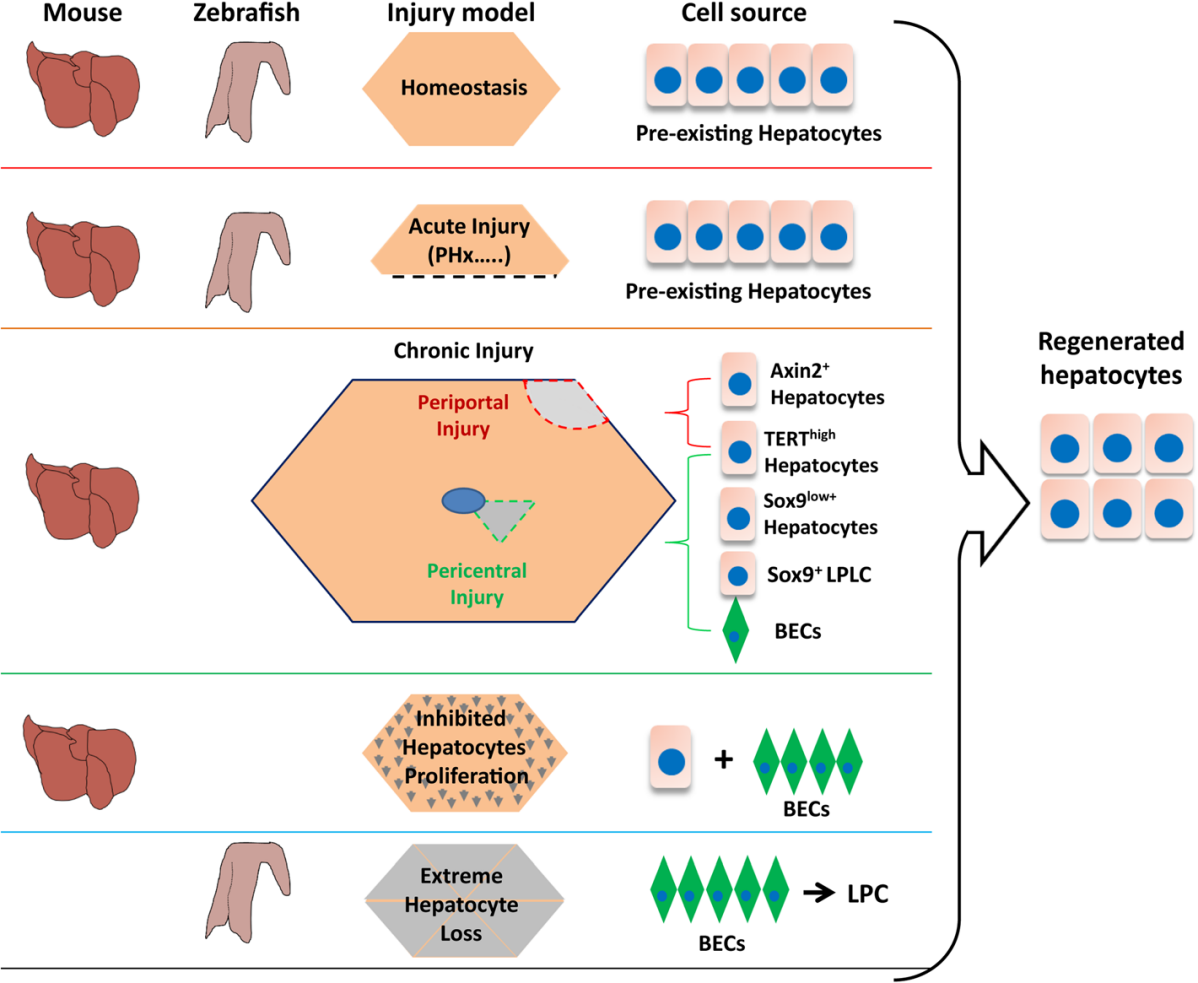
A cartoon for comparing origin of hepatocytes during liver homeostasis and regeneration in mouse and zebrafish. Liver initiates different regeneration modes in response to different degrees of damages. Proliferation of embryonic hepatocytes make main contribution to hepatocytes in a normal adult liver. Proliferation of mature hepatocytes are responsible for maintaining hepatocyte homeostasis in a normal adult liver and recovery of liver mass after acute injuries such as PH (top two panels). Chronic injuries near the periportal region (periportal injury) will probably activate the Axin2^+^ cells around central vein to produce new hepatocytes (highlighted in red, third panel). In contrast, chronic injuries around the central vein region (pericentral injury) seems to mobilize Sox9^low+^ or/and Sox9^+^ LPLC hepatocytes around periportal region to generate new hepatocytes (highlighted in green, third panel). As the degree of damage increases, such as treating mouse with TAA for 24 weeks, biliary epithelial cells (BECs) start to make contribution to the production of hepatocytes through direct lineage conversion (third panel). It is proposed that TERT^high^ hepatocytes might be a universal cell source for liver regeneration under periportal, pericentral or other injury conditions (third panel). Impaired proliferation ability of hepatocytes, such as in the case of *β1-integrin* knockdown or p21 overexpression, but not cell quantity loss, appears to act as the key cue for BECs activation (fourth panel). When > 95% hepatocytes were ablated in larva zebrafish, the organism can still survive by BECs-derived hepatocytes regeneration (bottom panel). Dashed line in the second panel: resection site; grey shaded area in hexagons along the third column: damage degrees; hepatocytes: pinkish cell with a large blue nucleus; biliary epithelial cells (BECs): green diamond with a small blue nucleus. LPC: liver progenitor cell

Numerous studies have been carried to explore the process and molecular mechanism under liver regeneration. Among many features related to liver regeneration, two aspects are widely appreciated: 1) it appears that the regeneration process and regulatory mechanisms are conserved across different species; 2) key developmental signaling pathways and certain key factors controlling liver organogenesis are reused during liver regeneration (Choi et al., 2014). In this review, we have focused on summarizing the origin of new hepatocytes during liver regeneration under different liver-damage models (Fig. 1). By taking into account of different regeneration responses triggered in different liver damage models in both zebrafish and mouse, we noticed some interesting features:

Firstly, if the damage models do not impede the capability of hepatocyte proliferation or do not ablate near-all hepatocytes it will lead to liver regeneration via hepatocyte proliferation (Fig. 1). Mature hepatocytes or polyploid hepatocytes have strong self-renewal capacity after several kinds of acute injury such as acute CCl_4_, CDE, AA, DDC treatment and PH (Yanger et al., 2014; Matsumoto et al., 2020; Chen et al., 2020a). Meanwhile, regional heterogeneity across each hepatic lobule may result in regeneration heterogeneity. Under the different damage model, periportal Sox9^+^ hepatocytes or TERT^High^ hepatocytes adjacent to sinusoid endothelial cells will make contribution to liver regeneration, respectively (Lin et al., 2018; Font-Burgada et al., 2015; Ding et al., 2010). The heterogeneity of hepatocytes during liver regeneration might be attributed to the polarized localization of hepatocytes within a liver which forms hepatic plates and liver lobule, creating different microenvironmental niches.

Secondly, if the injury models impair the proliferation activity of hepatocytes or ablate near-all hepatocytes it will leads to BEC dedifferentiation and LPC-to-hepatocyte differentiation mediated regeneration (Fig. 1), such is the case observed in zebrafish (Choi et al., 2014; He et al., 2014). Experiments of hepatocyte *β1-integrin* ablation and p21 overexpression in mouse model proved that impaired hepatocyte regeneration is required for cholangiocytes to form hepatocytes (Raven et al., 2017).

One remaining key question for liver regeneration is how the organisms sense the degree of liver loss and the degree of liver recovery. Although the molecular mechanisms involved are still unclear, recent study indicated that blood flow shear stress plays an important role (Lorenz et al., 2018). Liver injury will reduce the number of hepatic lobule and sinusoid, which leads to increased blood flow shear stress in the rest liver organ (Sato et al., 1999). Since hepatic lobule is the functional unit of liver and is functionally heavily dependent on sinusoid, (Frevert et al., 2005) we have reason to propose that the organisms control liver lobule number by blood vessel and blood flow. In view of this, the relative stability of LBR (liver to body ratio) may represent the balance between liver function and metabolic demand of the body, which reflected by the blood flow shear stress. Answer to this question will no doubt advance our understanding of the full picture of liver regeneration [70].
